# Drug Repurposing and Polypharmacology to Fight SARS-CoV-2 Through Inhibition of the Main Protease

**DOI:** 10.3389/fphar.2021.636989

**Published:** 2021-02-22

**Authors:** Luca Pinzi, Annachiara Tinivella, Fabiana Caporuscio, Giulio Rastelli

**Affiliations:** ^1^ Molecular Modelling and Drug Design Lab, Life Sciences Department, University of Modena and Reggio Emilia, Modena, Italy; ^2^ Clinical and Experimental Medicine PhD Program, University of Modena and Reggio Emilia, Modena, Italy

**Keywords:** COVID-19, SARS-2-CoV-2, drug repurposing, polypharmacology, structure-based, molecular docking, BEAR

## Abstract

The outbreak of a new coronavirus (SARS-CoV-2), which is responsible for the COVID-19 disease and is spreading rapidly around the world, urgently requires effective therapeutic treatments. In this context, drug repurposing represents a valuable strategy, as it enables accelerating the identification of drug candidates with already known safety profiles, possibly aiding in the late stages of clinical evaluation. Moreover, therapeutic treatments based on drugs with beneficial multi-target activities (polypharmacology) may show an increased antiviral activity or help to counteract severe complications concurrently affecting COVID-19 patients. In this study, we present the results of a computational drug repurposing campaign that aimed at identifying potential inhibitors of the main protease (M^pro^) of the SARS-CoV-2. The performed *in silico* screening allowed the identification of 22 candidates with putative SARS-CoV-2 M^pro^ inhibitory activity. Interestingly, some of the identified compounds have recently entered clinical trials for COVID-19 treatment, albeit not being assayed for their SARS-CoV-2 antiviral activity. Some candidates present a polypharmacology profile that may be beneficial for COVID-19 treatment and, to the best of our knowledge, have never been considered in clinical trials. For each repurposed compound, its therapeutic relevance and potential beneficial polypharmacological effects that may arise due to its original therapeutic indication are thoroughly discussed.

## Introduction

At present, we are faced with one of the most devastating pandemic crises in human history, the coronavirus disease 2019 (COVID-19), which has affected around 56 million people to date and is responsible for more than 1.38 million deaths (Who.int^1^). Unfortunately, effective therapeutic options are not currently available to prevent or cure this disease, which is caused by the severe acute respiratory syndrome coronavirus 2 (SARS-CoV-2) (Who.int^1^). Only very recently, Pfizer, in partnership with BioNTech Manufacturing GmbH, has filed their clinically evaluated vaccine (Comirnaty) to Food and Drug Administration (FDA), for an Emergency Use Authorization (EUA) (Pfizer Inc.^2^) and received a Conditional Marketing Authorization from the European Medicines Agency (EMA^3^). However, it should be noted that the efficacy of vaccines for this disease have yet to be assessed on a large scale, and challenges in vaccine dose supply worldwide should be overcome. Moreover, the approval by worldwide regulatory agencies of effective and un-restricted treatments for patients already suffering from the disease is urgently needed.

SARS-CoV-2 is a positive-sense single-stranded RNA virus belonging to the β-genus of the *Coronaviridae* family (Wan et al., 2020) and is closely related to coronaviruses responsible for the SARS and MERS outbreaks that spread in 2003 and 2012, respectively (Zhu et al., 2020). Although outbreaks deriving from coronavirus infections appear to be recurrent (Zhu et al., 2020), no specific antiviral drugs are currently available to treat these diseases. Therefore, major efforts are currently focused on developing vaccines, as well as effective drugs to treat infected patients (Clinicaltrials.gov ^4^). Indeed, the first COVID-19 vaccine has been approved after the submission of the present study. In the meanwhile, many biological targets are now under investigation to develop SARS-CoV-2-specific antiviral compounds, the most studied being the RNA-dependent RNA polymerase, the spike protein, and the main protease (Morse et al., 2020). Moreover, the number of clinical trials based on antivirals developed for other infections or drugs meant to reduce inflammation and severe respiratory complications are now increasing at previously unseen rates (Clinicaltrials.gov ^4^). Currently investigated antivirals include favipiravir, an anti-*influenza* drug, which resulted effective also against the Ebola virus (ClinicalTrials Identifiers: NCT04303299, NCT04310228, NCT04349241), and the RNA-dependent RNA polymerase prodrug inhibitor remdesivir, which was shown to be effective also against MERS and SARS (Clinicaltrials.gov)^5^. Indeed, remdesivir has very recently been reported to significantly reduce the median recovery time of COVID-19 patients and has been approved by FDA as a therapeutic option in hospital settings (Beigel et al., 2020). Moreover, boceprevir, which is a NS3/4A serine protease inhibitor used in the treatment of chronic Hepatitis C, has also very recently gained the attention of the research community for COVID-19 treatment (Fu et al., 2020). Hydroxychloroquine was also investigated for the treatment of COVID-19, but clinical trials on this compound were stopped by the World Health Organization in June 2020 (Who.int^6^). Moreover, hydroxychloroquine did not show significant activity on COVID-19 hospitalized patients, according to recently retrieved clinical data (RECOVERY Collaborative Group et al., 2020b). The monoclonal antibodies tocilizumab and sarilumab directed against the interleukin-6 receptor (IL-6R) have entered clinical trials (ClinicalTrials Identifiers: NCT04322773, NCT04332913) for the same purpose. Finally, the use of dexamethasone was endorsed by the European Medicines Agency to treat hospitalized patients with COVID-19 (RECOVERY Collaborative Group et al., 2020a).

The rapid spread of the SARS-CoV-2 pandemic and the lack of specific antiviral drugs suggest that drug repurposing should be the preferred way for rapidly selecting suitable candidates for clinical testing (Senanayake, 2020). Indeed, these compounds are well characterized and already possess optimized pharmacokinetics and safety profiles. In this scenario, computational approaches can provide new opportunities for drug repurposing (March-Vila et al., 2017), allowing the identification of valuable drug candidates to be used either alone or in combination. These approaches take advantage of curated databases reporting chemical, structural and activity information on different protein targets and already known therapeutic effects (e.g., PubChem, ChEMBL, DrugBank), or other repositories specifically focusing on repurposing data (e.g., ReframeDB and Drug Repurposing Hub) and COVID-19 databases (e.g., NIH^7^), which are currently available for already approved or under clinical evaluation COVID-19 drugs (Corsello et al., 2017; Gaulton et al., 2017; Janes et al., 2018; Wishart et al., 2018; Kim et al., 2019). Moreover, considering the severe life-threatening disease complications, polypharmacological drugs, *i.e.*, single drug molecules that combine antiviral activity with e.g., anti-inflammatory or antithrombotic activity, may be particularly useful (Anighoro et al., 2014). To this end, in this study we performed an extensive structure-based virtual screening campaign and identified 22 top-candidate approved or experimental drugs as potential inhibitors of the SARS-CoV-2 main protease (M^pro^). The DrugBank database (Wishart et al., 2018), including drug metabolites, was docked to the M^pro^ enzyme and the results were post-processed with BEAR (Rastelli et al., 2009), an *in-house* developed screening tool with a well-documented ability to refine virtual screening results (Rastelli and Pinzi, 2019). Moreover, retrospective structure-based analyses were performed on a set of compounds with already known SARS-CoV-2 M^pro^ activity, to further validate the adopted *in silico* structure-based protocol. The candidate selection process took into special consideration the analysis of drug annotations and biological activity information reported in the literature to attain possible favorable polypharmacological effects arising from the original therapeutic indication. Because of their fitting to the SARS-CoV-2 main protease active site, the reported drugs could be readily repurposed to elicit an antiviral response.

## Materials and Methods

The recently reported 6LU7 crystal structure of the SARS-CoV-2 main protease (Jin et al., 2020) was first collected from the Protein Data Bank (accessed on March 17th, 2020) and then prepared for the *in silico* screening process using the Protein Preparation Wizard (Protein Preparation Wizard, Schrödinger, LLC, New York, NY, 2020). Defaults parameters were used during the protein preparation. Atom types and connectivity issues were fixed, hydrogen atoms were added, and interaction geometries were optimized. The co-crystallized water molecules and the peptide-like PRD_002214 inhibitor (compound N3) were retained during the protein preparation process, while they were removed in the following docking and post-processing phases.

Docking calculations were performed in the active site of the prepared 6LU7 crystal structure by using Glide (Glide, Schrödinger, LLC, New York, NY, 2020; Friesner et al., 2004) with the Standard Precision (SP) protocol. Specifically, the receptor grid was first generated on the coordinates of the co-crystallized PRD_002214 ligand, with a box of (10 Å × 10 Å × 10 Å) dimensions (default settings). Then, the docking protocol was validated by redocking the co-crystallized ligand into its parent crystal structure, with satisfactory results.

Approved drugs, clinical and preclinical candidates, and metabolites were first downloaded from the DrugBank database (www.drugbank.ca, accessed on March 17th, 2020), and then prepared for the structure-based calculations using the LigPrep utility (LigPrep, Schrödinger, LLC, New York, NY, 2020). Specifically, ionization states and tautomers at pH values equal to 7 ± 2 were first generated for each ligand in the screening database and then minimized according to the OPLS3e force field. Stereoisomers were also generated for the DrugBank ligands with undefined chiralities. Subsequently, the pre-treated compounds were screened with the validated docking protocol. Finally, the predicted poses were visually inspected, and the first 2000 top-scoring ligand-protein complexes were further post-processed with the BEAR post-docking tool (Rastelli et al., 2009).

The BEAR protocol consists of three steps based on molecular mechanics (MM) minimization and molecular dynamics cycles, followed by more accurate binding free energy estimation of the refined complex with the MM-PBSA and MM-GBSA methods (Rastelli et al., 2009). Further details on the BEAR post-processing procedure, which was shown to considerably increase the prediction performances in several virtual screening campaigns, are reported by (Rastelli et al., 2009).

A final step of visual inspection of the refined complexes and their comparison with the corresponding poses predicted by Glide aided in the final selection of the potential candidates for the SARS-CoV-2 main protease inhibition. Analysis of data annotation and literature searches returned a set of top candidates with a potentially beneficial polypharmacology profile.

Retrospective structure-based calculations were also performed with the previously described protocol on a set of compounds (Supplementary Table S1) with recently reported activity data on the SARS-CoV-2 M^pro^ (Dai et al., 2020; Jin et al., 2020; Ma et al., 2020). Finally, results were visually inspected and the obtained scores compared with those from the performed virtual screening.

## Results

The SARS-CoV-2 main protease (M^pro^), also known as 3C-like protease, is a relevant target for drug repurposing because it plays a crucial role in the maturation of the viral particle (Jin et al., 2020). Indeed, this cysteine protease presents a highly conserved active site in several coronaviruses, such as SARS-CoV and MERS-CoV, and plays a key role in the cleavage of precursor polyproteins translated from viral RNA.

**FIGURE 1:**
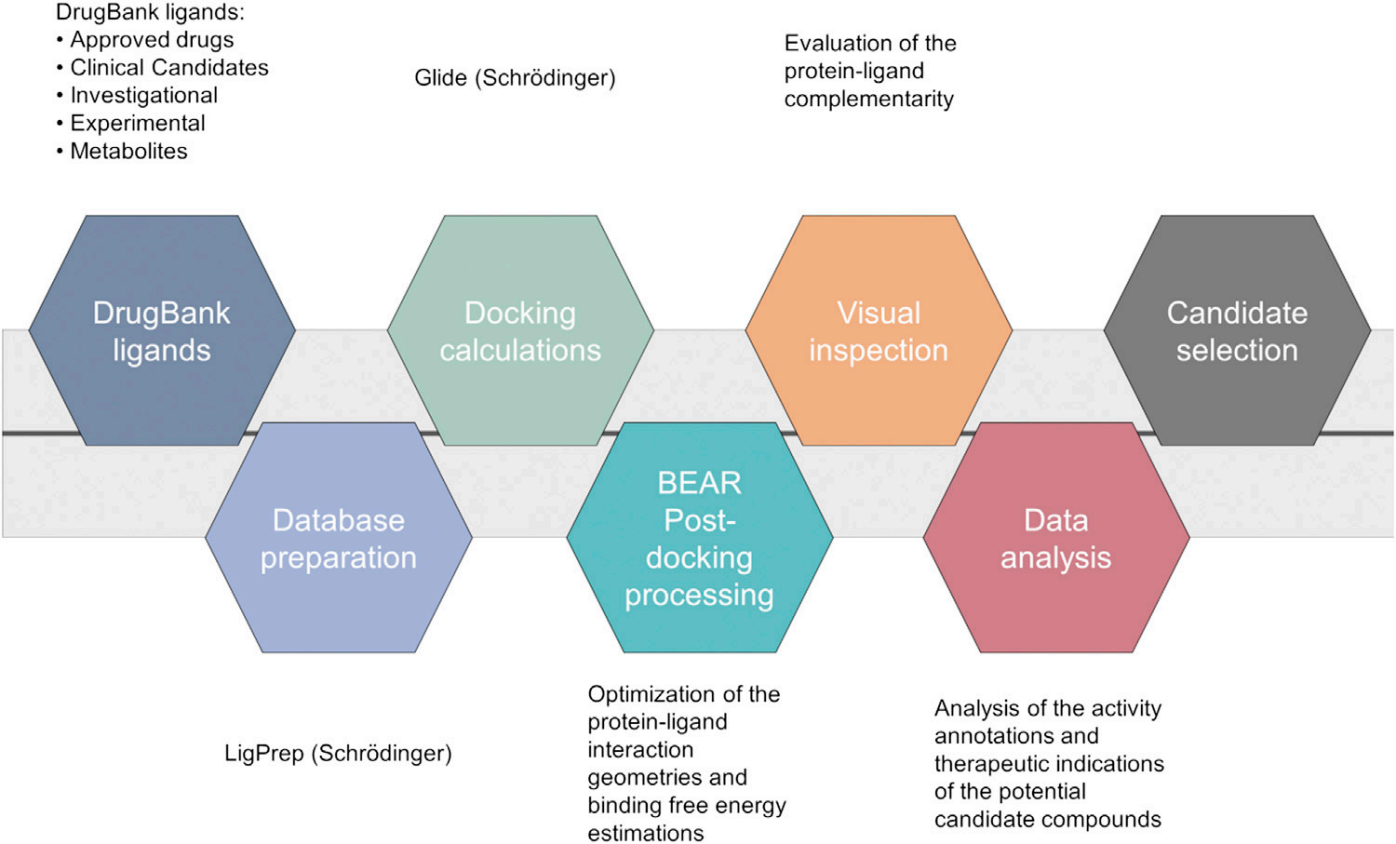
Computational workflow of the repurposing screening for the identification of SARS-CoV-2 main protease inhibitors.

A computational screening workflow (Figure 1) was devised to identify drug candidates able to bind and inhibit the SARS-CoV-2 main protease. To this end, the complete DrugBank database (Release Version 5.1.5, 13,227 compounds, accessed on March 17th, 2020), including drug metabolites, was docked to the crystal structure of the SARS-CoV-2 main protease (PDB ID: 6LU7) (Jin et al., 2020) using Glide (Glide, Schrödinger, LLC, New York, NY, 2020; Friesner et al., 2004), and the results were ranked according to the Glide “*Docking score*” scoring function.

The 2000 top ranking compounds were post-processed with BEAR (Rastelli et al., 2009; Rastelli and Pinzi, 2019). BEAR is an automated procedure that combines the structural refinement of docking poses through molecular dynamics, which accounts for protein flexibility, with the re-ranking of ligands on the basis of binding free-energies. Binding free energies of the refined complexes are calculated through the molecular mechanics generalized Born surface area (MM-GBSA) and molecular mechanics Poisson Boltzmann surface area (MM-PBSA) scoring functions, which implicitly account for solvation effects (Rastelli et al., 2009). Then, the best candidates were selected according to i) docking and post-docking scores, which provide an estimation of the binding affinity of a ligand to a target, ii) visual inspection of the protein-ligand complexes, and iii) analysis of drug annotations and literature information to repurpose known drugs, clinical candidates or experimental compounds as M^pro^ inhibitors that may benefit from their original therapeutic indications to reduce severe COVID-19 complications, while inhibiting viral particle maturation (beneficial polypharmacology (Anighoro et al., 2014)). Interestingly, several ligands already present in the DrugBank database, which were very recently confirmed as inhibitors of the SARS-CoV-2 M^pro^ enzyme, turned out to be among the top-scored compounds in our virtual screening campaign (see Supplementary Table S1), thus demonstrating that the used protocol is able to identify active compounds within the screened database. Moreover, to further validate the ability of the used *in silico* workflow to aid in the identification of candidates with potential SARS-CoV-2 M^pro^ activity, retrospective analyses were also performed on a set of inhibitors extracted from the literature and not included in the DrugBank database. In particular, ligand structures were first collected from their reference publications (Dai et al., 2020; Jin et al., 2020; Ma et al., 2020), built and prepared for structure-based calculations. Then, docking calculations followed by BEAR binding-free energy estimations were performed, as detailed in the “Materials and Methods” section. Notably, the majority of retrospectively investigated compounds with known activity data (i.e., 16 out of 23; ∼70% of the validation dataset) provided comparable docking scores to those of the 2,000 top ranking compounds (Glide “*Docking scores*” lower than −7.18 Kcal/mol), therefore, these compounds would have been selected for further investigations in our workflow. Moreover, it should be noted that the seven known SARS-CoV-2 M^pro^ inhibitors discarded at this stage by our workflow present a chemical structure significantly different to that of the crystallographic ligand, and it is widely acknowledged that docking results can be strongly affected by the selected receptor conformation (Broccatelli and Brown, 2014). Finally, results of the BEAR calculations on the retrospectively investigated compounds further strengthen the soundness of the implemented workflow. Indeed, the most active compounds in the validation dataset provided BEAR GB and PB scores comparable to those of the best-ranking ligands in the screened database (Supplementary Table S1).

The most promising compounds resulting from the virtual screening are reported in Table 1, along with their DrugBank IDs, the predicted docking and binding free energy scores, the rank position in the screening outcome, the therapeutic targets and original indications, and the PDB IDs of the crystal structure of each drug in complex with its original target, when available. The 2D chemical structures of the selected candidates are reported in Supplementary Material S1. A detailed discussion of the selected drug candidates divided into candidates for drug repurposing, candidates for drug repurposing with beneficial polypharmacology, and candidates for drug repurposing based on active drug metabolites, is reported in the following paragraphs.

**TABLE 1:** Drug candidates selected from the computational repurposing campaign.

DrugBank ID	Compound name	Scores (kcal/mol)			Rank #			PDB codes	Primary targets (UNIPROT ID)	Therapeutic indications
		Glide	BEAR GB	BEAR PB	Glide	BEAR GB	BEAR PB			
Candidates for drug repurposing										
DB01871	Cruz-1; EXPT02989	−9.3	−62.2	−39.7	90	206	76	1ME4	Cathepsin F (Q9UBX1); cruzipain (P25779)	Chagas disease (experimental)
DB02128	Cruz-2; EXPT02467	−9.4	−62.7	−40.4	82	187	65	1ME3	Cruzipain (P25779)	Chagas disease (experimental)
DB02378	MMI-175; EXPT02196	−12.0	−82.7	−62.5	3	20	1	1XS7	β-Secretase 1 (P56817)	Alzheimer’s disease (experimental)
DB03063	EH58; EXPT01332	−12.2	−65.4	−46.4	2	129	21	1LF3	Plasmepsin-2 (P46925)	Malaria (experimental)
DB03648	EXPT00713	−11.2	−73.2	−40.0	8	48	72	1RL4	Formylmethionine deformylase (Q8I372)	Malaria (experimental)
DB04353	QF34; EXPT02729	−10.0	−60.9	−39.7	35	229	77	1IZH; 1IZI	HIV-1 protease (O90777)	HIV-1 infection (experimental)
DB04378	RS370; EXPT02746	−9.0	−50.6	−28.9	121	612	511	1LF2	Plasmepsin-2 (P46925)	Malaria (experimental)
DB04502	WRR-204; EXPT03235	−9.0	−63.8	−33.1	118	165	257	1EWO	Cruzipain (P25779)	Chagas disease (experimental)
DB04595	N3; PRD_002214	−9.9	−58.4	−35.8	42	292	151	6LU7	SARS-CoV-2 main protease	SARS-CoV-2 infection (experimental)
DB04692	I2	−10.2	−69.8	−49.0	25	74	11	2D2D	3C-like proteinase (P0C6X7)	SARS-CoV infection (experimental)
DB04710	N1	−9.1	−64.0	−34.3	117	159	195	1WOF; 2AMP	3C-like proteinase (P0C6X7)	SARS-CoV infection (experimental)
DB08732	WRR-183	−9.5	−52.1	−29.9	65	526	441	2OP9	SARS-CoV replicase polyprotein 1a (P0C6U8); SARS-CoV replicase polyprotein 1 ab (P0C6X7)	SARS-CoV infection (experimental)
DB11938	Difelikefalin	−11.1	−77.9	−34.0	9	32	217		κ-Opioid receptor (P41145)	Acute pain and post-operative pain treatment (investigational)
Candidates for drug repurposing with beneficial polypharmacology										
DB03395	Enalkiren	−9.7	−67.9	−45.3	56	89	27		Renin (P00797)	Agent acting on the renin-angiotensin system^b^
DB04653	Calpain inhibitor IV; ZLLYCH_2_F	−9.7	−66.9	−42.5	52	100	44	1ZCM	Calpain-1 catalytic subunit (P07384)	Aging and ageing-related diseases (experimental)
DB04758	Ethylsulfonamide-D-Trp-Gln-p-aminobenzamidine	−10.6	−66.6	−44.1	17	106	1,581	1WUN	Coagulation factor VII (^087^P09)	Anticoagulant (experimental)
DB07571	Z-LY-CMK	−9.2	−53.7	−29.6	99	444	464	2FZS	ATP-dependent Clp protease proteolytic subunit (P0A6G7)	Potential antimicrobial agent (experimental)
DB07934	BM51.1011	−8.8	−66.4	−49.3	155	111	10	1UVS	Prothrombin (P00734)	Anticoagulant (experimental)
DB12955	Delparantag; PMX-60056	−13.1	−108.7	−48.1	1	1	14		Heparin antagonist^c^	Angioplasty, coronary artery disease, percutaneous coronary intervention (experimental)
Candidates for drug repurposing based on active drug metabolites										
DBMET00084	Ritonavir M7^a^	−7.6	−62.5	−49.7	935	194	9		HIV-1 protease (O90777)	HIV-1 infection^a^
DBMET01548	Saquinavir M10^a^	−10.5	−66.0	−38.9	20	119	90		HIV-1 protease (O90777)	HIV-1 infection^a^
DBMET01549	Saquinavir M2	−10.5	−67.7	−40.2	19	92	68		HIV-1 protease (O90777)	HIV-1 infection^a^
DBMET01550	Saquinavir M2	−10.5	−62.6	−31.8	18	192	320		HIV-1 protease (O90777)	HIV-1 infection^a^

Compound DB04595, which is a SARS-CoV-2 M^pro^ inhibitor, emerged in a previous computational screening and is shown in italics.

For metabolites, therapeutic targets and indications of their parent drugs are shown.

Glide (“Docking Score”), BEAR GB (binding free-energy estimation through the MM-GBSA method) and BEAR PB (binding free-energy estimation through the MM-PBSA method) values were used to estimate the ligand-protein binding affinity (values are reported in kcal/mol).

Bibliographic references of the crystal structures are reported in Supplementary Table S2.

^a^ Data retrieved from: ChEMBL, accessed on April 20th, 2020.

^b^ Data retrieved from: https://www.drugs.com, accessed on April 20th, 2020.

^c^ Data retrieved from: KEGG, accessed on April 23rd, 2020.

### Candidates for Drug Repurposing

The drugs herein reported (either approved or investigational) are excellent examples of potential candidates for repurposing as SARS-CoV-2 M^pro^ inhibitors. Indeed, all compounds are well accommodated in the SARS-CoV-2 M^pro^ catalytic site. Moreover, as evidence of the reliability of our results, several of the proposed candidates are viral protease inhibitors, which in some cases are already under study as COVID-19 therapeutic agents (see below).

The hydroxymethyl ketones EXPT02467 (cruz-2, DB02128) and EXPT02989 (cruz-1, DB01871) are two reversible inhibitors of cruzipain, a cysteine-type endopeptidase of *Trypanosoma cruzi* (Cazzulo et al., 1990). The structures of their complexes with the cruzipain target are available under the PDB IDs 1ME3 and 1ME4, respectively. Another interesting cruzipain inhibitor that emerged from our screening is WRR-204 (EXPT03235, DB04502), which is an irreversible inhibitor (PDB ID: 1EWO). To the best of our knowledge, these compounds have never been investigated for drug repurposing, including on COVID-19 related therapeutic targets.

**FIGURE 2:**
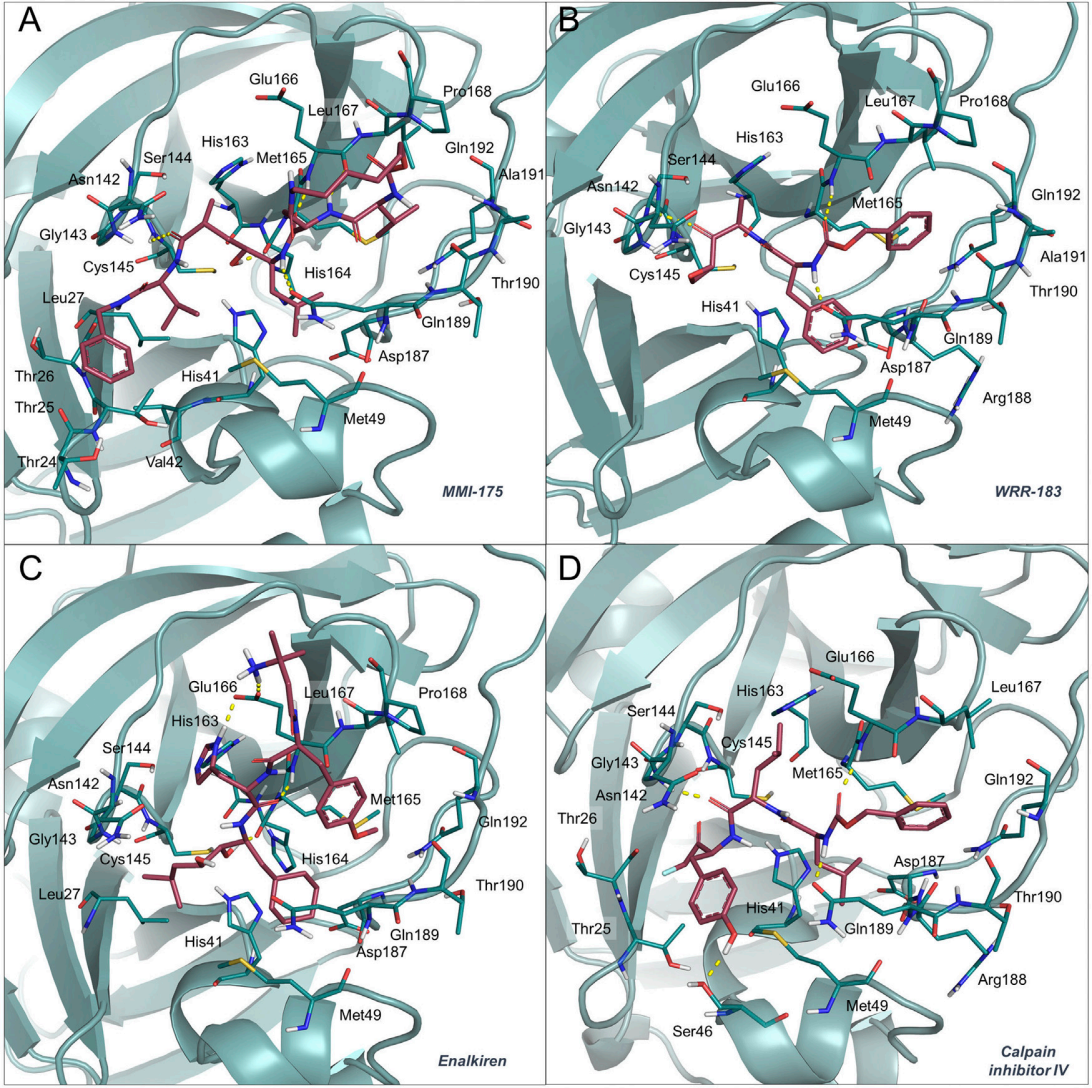
Binding mode of MMI-175 **(A)**, WRR-183 **(B)**, enalkiren **(C)** and calpain inhibitor IV **(D)** to the 6LU7 crystal structure. The SARS-CoV-2 M^pro^ binding site residues and the potential repurposed compounds are represented in deepteal and raspberry sticks, respectively. The image was created with PyMol (The PyMOL Molecular Graphics System, Version 2.1.1, Schrödinger, LLC).

MMI-175 (DB02378) is an experimental drug that inhibits β-secretase (BACE-1) (Ghosh et al., 2005), one of the two aspartic proteases responsible for the generation of amyloid-β peptides in neurons. As such, drugs blocking this enzyme may aid in slowing down Alzheimer’s disease progression. According to the predicted docking pose (Figure 2, panel (A)) and binding affinities, this compound is expected to efficiently bind to the SARS-CoV-2 M^pro^ enzyme. The ability of this compound to cross the blood-brain barrier would be of great interest for COVID-19 treatment, as previous studies have reported the presence of coronavirus particles in the CNS and their potential association with neurologic manifestations in patients (Baig et al., 2020). Also in this case, the compound emerged for the first time as a putative candidate for drug repurposing, according to the available literature data.

EH58 (EXPT01332, DB03063) is a potent inhibitor of plasmepsin 2, an aspartic protease in the food vacuole of *Plasmodium falciparum* (PDB ID: 1LF3), and exhibits antimalarial activity (K_i_ = 100 nM) (Asojo et al., 2003). Another plasmepsin 2 inhibitor worthy of consideration, even if predicted scores are lower than those of EH58, is RS370 (DrugBank ID: DB04378, PDB ID: 1LF2), with a reported K_i_ of 30 nM on its original target (Asojo et al., 2002). EXPT00713 (DB03648) is a *P. falciparum* formylmethionine deformylase inhibitor with a reported IC_50_ of 130 nM (Robien et al., 2004). Interestingly, several antimalarial drugs are under the spotlight of COVID-19 investigations (Gendrot et al., 2020). The possibility of repurposing these compounds for the COVID-19 treatment would provide great benefits for cases of malaria and SARS-CoV-2 co-infections. This is especially important in those countries where malaria is endemic, considering that COVID-19 early symptoms (e.g., fatigue and fever) might result in misleading clinical diagnoses (Di Gennaro et al., 2020).

QF34 (EXPT02729, DB04353) is a pseudopeptide inhibitor of several variants of HIV-1 and HIV-2 proteases (Weber et al., 2002), including those of some highly resistant mutants. The compound was also crystallized with the HIV-1 protease (PDB IDs: 1IZH, 1IZI). Interestingly, the identification of ligands with already optimized safety profiles and reported HIV activity might be particularly effective for HIV/SARS-CoV-2 co-infected patients, which, unfortunately, seem to have poorer prognoses (Bhaskaran et al., 2020).

Compounds I2 (DB04692), N1 (DB04710) and N3 (PRD_002214, DB04595), three experimental inhibitors of the SARS-CoV M^pro^ reported in 2005, may also be valuable candidate inhibitors of the SARS-CoV-2 M^pro^. Compounds I2, N1, and N3 were co-crystallized in complex with the SARS-CoV M^pro^ (PDB IDs: 2D2D, 1WOF, and 2AMQ, respectively). Notably, compound N3 has been very recently confirmed to bind and inhibit the SARS-CoV-2 M^pro^, and the crystal structure of its complex was the starting point of our virtual screening (PDB ID: 6LU7) (Jin et al., 2020).

Compound WRR-183 (DB08732) is an α,β-epoxyketone that irreversibly inhibits the SARS-CoV M^pro^ (PDB ID 2OP9) (Goetz et al., 2007). According to our docking results, this compound was predicted to bind to the SARS-CoV-2 M^pro^ tightly and with a similar binding mode, where the epoxide is in close proximity to the Cys residue (Figure 2, panel (B)). Moreover, WRR-183 and especially its C-2 (R) epoxide isomer WRR-182 are highly active against the SARS-CoV spike-mediated entry (Zhou et al., 2011). Therefore, they show the potential to block SARS-CoV and, hopefully, SARS-CoV-2 at two different steps of the replication cycle, *i.e.,* viral entry and particle assembly (Zhou et al., 2011).

Difelikefalin (formerly known as CR-845, DB11938) is a highly selective agonist of the κ-opioid receptor (Keppel Hesselink, 2017). This analgesic opioid peptide acts peripherally and is under investigation for the treatment of acute and post-operative pain and, more recently, chronic pruritus (Keppel Hesselink, 2017). Difelikefalin is currently in two Phase II clinical trials for the treatment of pruritus in atopic dermatitis and biliary cholangitis (Clinical Trials Identifiers: NCT04018027 and NCT03995212). The peripheral analgesic activity of the compound, together with its potential SARS-CoV-2 M^pro^ activity, may prove beneficial to COVID-19 patients experiencing peripheral neurologic symptoms and pain.

### Candidates for Drug Repurposing With Beneficial Polypharmacology

Polypharmacological ligands are extremely interesting in drug repurposing, because they offer the potential for higher efficacy and a combination of synergistic effects (Anighoro et al., 2014). Therefore, for each top-ranking compound, we carefully investigated whether a possible beneficial polypharmacological effect may arise owing to the reported biological activities and original therapeutic indications.

Enalkiren (DB03395) belongs to the class of direct renin inhibitors. By mimicking the transition state of angiotensin, enalkiren is able to block the first step of the renin-angiotensin system (Glassman et al., 1990). Interestingly, hypertension is among the most common comorbidities in hospitalized COVID-19 patients, and is often treated with angiotensin II receptor blockers or angiotensin-converting enzyme (ACE) inhibitors (Ran et al., 2020). Unfortunately, it has been recently reported that SARS-CoV-2 binds to the widespread angiotensin-converting enzyme 2 receptor to enter target cells (Bavishi et al., 2020) and that levels of serum angiotensin II are considerably increased in COVID-19 patients (Liu et al., 2020b). Moreover, the use of drugs lowering blood pressure through such mechanisms might lead to overexpression of ACE2, thus potentially increasing the susceptibility to SARS-CoV-2 in patients (Fang et al., 2020). Based on these premises, the possibility of enalkiren to modulate the renin-angiotensin system without altering patient susceptibility to the virus, together with inhibition of the SARS-CoV-2 M^pro^, may exhibit beneficial effects for COVID-19 treatment. According to our docking results, enalkiren is well accommodated within the SARS-CoV-2 M^pro^ binding site (Figure 2, panel (C)). Interestingly, another very recent computational study based on a different workflow also identified enalkiren as a potential candidate to target the SARS-CoV-2 M^pro^, further supporting its selection as a promising candidate for COVID-19 treatment (Liu et al., 2020a).

The calpain inhibitor IV (ZLLYCH_2_F, DB04653) is a covalent inhibitor of the calpain-1 cysteine protease (PDB ID: 1ZCM) that regulates the activity of proteins involved in processes influencing neuronal plasticity, cognition and neurodegeneration (Vosler et al., 2008) and is a potential target for intervention in inflammatory diseases (Cuzzocrea et al., 2000). In our docked structure (Figure 2, panel (D)), the reactive methylene group of the compound is in close proximity to the Cys residue of the SARS-CoV-2 M^pro^ active site. This result indicates that a covalent bond can potentially be formed that would provide specificity and higher affinity over other proteases. Interestingly, in 2004, calpain inhibitor IV was shown to be an active agent against SARS-CoV (Barnard et al., 2004) and its potential use against SARS-CoV-2 has also very recently been proposed in other repurposing studies (Abhithaj et al., 2020). Moreover, other calpain inhibitors have recently been tested for inhibition of the SARS-CoV-2 M^pro^ and among them the calpain inhibitor XII was shown to be the most active with an IC_50_ of 0.45 μM (Ma et al., 2020). Importantly, calpain inhibitor IV is reported to also act as an inhibitor of cathepsin L (Angliker et al., 1992), which is a necessary factor for the SARS-CoV-2 entry into host cells (Ou et al., 2020). Based on these premises, calpain inhibitor IV represents a valuable multi-target candidate for further clinical investigation to treat COVID-19.

Ethylsulfonamide-D-Trp-Gln-p-aminobenzamidine (DB04758) was designed to potently inhibit factor VIIa (FVIIa), which forms a complex with tissue factor (TF) to initiate the extrinsic coagulation cascade (Kadono et al., 2005). Compared with other anti-thrombotic agents, the specific targeting of the extrinsic coagulation provides a decreased risk of bleeding. SARS-CoV-2 infection often has dramatic consequences for the circulatory system (Tang et al., 2020b), with preliminary reports including thrombocytopenia, elevated d-dimer levels, prolonged prothrombin time, and disseminated intravascular coagulation (Han et al., 2020). Interestingly, research findings suggest that inhibition of TF-FVIIa complex may reduce the cytokine storm responsible for the increased coagulation and multi-organ failure, and thus the mortality rate in COVID-19 patients (Geisbert et al., 2003; Eslamifar et al., 2020). Our *in silico* findings suggested that DB04758 could also bind with high affinity to the SARS-CoV-2 M^pro^. Therefore, this molecule might exhibit a dual activity against two crucial aspects of the SARS-CoV-2 infection.

Z-LY-CMK (DB07571) is a covalent inhibitor of the ATP-dependent Clp protease proteolytic subunit (ClpP), an enzyme that has recently gained attention as a promising drug target for antibiotic development (Bhandari et al., 2018). If confirmed, the potential of this compound to act as both an antimicrobial agent and a SARS-CoV-2 inhibitor would be particularly useful to treat secondary bacterial infections, potentially affecting COVID-19 patients. Moreover, a structurally similar compound (Z LVG CHN2 in the original paper), previously shown to inhibit the herpes simplex virus cysteine protease (Björck et al., 1990), has recently been tested on Vero E6 cells infected with SARS-CoV-2. Its antiviral activity (EC_50_ = 0.19 µM) was suggested to be due to the inhibition of the SARS-CoV-2 M^pro^ (Riva et al., 2020).

The thrombin inhibitor BM51.1011 (DB07934) is another candidate with a promising multi-target activity profile. Indeed, this compound has been originally studied as a low molecular weight inhibitor of thrombin, a class of therapeutics with anticoagulant effects (Engh et al., 1996). The structure of BM51.1011 in complex with the original target (thrombin) is reported in PDB ID: 1UVS. Notably, thrombotic complications and, in particular, coagulopathies appear to be an important issue in patients affected by COVID-19 (Han et al., 2020). Moreover, reduced levels of antithrombin, a protein involved in the regulation of the coagulation cascade, have been reported in COVID-19 patients (Arachchillage et al., 2020). Interestingly, according to recent findings, treatment with Argatroban, a direct thrombin inhibitor with anticoagulant effects independent from antithrombin, seems to provide significant therapeutic effects in patients (Arachchillage et al., 2020; Sagardia and Daniels, 2020), albeit this drug was shown to be unable to inhibit SARS-CoV-2 replication (NIH^8^). As the pandemic is spreading, the reported coagulation disorders in COVID-19 patients as in previous SARS and MERS patients should be carefully addressed. Based on these premises, the possibility of BM51.1011 to exert anticoagulant and M^pro^ inhibitory activity, if confirmed, would certainly provide a valuable therapeutic advantage for COVID-19 patients.

Delparantag (formerly known as PMX-60056, DB12955) is a top scoring candidate for all the scoring functions. This molecule reverses the anticoagulation effects of heparin by binding to the pentasaccharide group of unfractionated heparin (UFH) and low-molecular-weight heparins (LMWH) (Kuziej et al., 2010). Heparin has gained increasing attention for its ability to prevent blood coagulation in COVID-19 patients affected by severe pneumonia and its concomitant anti-inflammatory effects that result in reduced IL-6 levels (Tang et al., 2020a). However, recent studies have shown that patients with COVID-19 can rapidly develop severe or critical vascular diseases, which may result in venous thromboembolism and bleeding status (Xu et al., 2020). As a close monitoring of venous thromboembolism and bleeding risks is essential in such patients, delparantag may be an effective tool to mitigate bleeding risks while eliciting antiviral activity due to the potential inhibition of the SARS-CoV-2 M^pro^. To the best of our knowledge, this compound has never been investigated in repurposing campaigns.

### Candidates for Drug Repurposing Based on Active Drug Metabolites

A unique feature of our repurposing strategy involves the inclusion of drug metabolites among screened compounds. Although they are typically discarded in drug repurposing studies, major metabolites can provide extremely interesting results. For this reason, here we describe some of the top scoring metabolites from our screening. Remarkably, many of these metabolites were shown to bind to the active site of the SARS-Cov-2 M^pro^ with higher affinity than their parent drug.

Ritonavir and lopinavir, two HIV-1 protease inhibitors that are administered in combination, are currently being clinically investigated against COVID-19 (Cao et al., 2020). Unfortunately, recent research findings showed that lopinavir or its combination with ritonavir are not able to significantly inhibit the SARS-CoV-2 M^pro^ up to a concentration of 100 µg/m (Jang et al., 2020). However, they were shown to be slightly active on other SARS-CoV-2 related targets, as reported in the COVID-19 open-data portal of the National Institutes of Health (NIH^9^; NIH^10^). Based on our *in silico* analyses, neither of these drugs appears to interact favourably with the SARS-CoV-2 M^pro^, although the N-desmethyl metabolite M7 of ritonavir (DBMET00084) was demonstrated to better interact with the protein owing to its free urea group that establishes favourable hydrogen bonds with the SARS-CoV-2 M^pro^.

The saquinavir decahydroisoquinoline metabolites M2 (DBMET01550 and DBMET01549) and t-butyl hydroxyl M10 (DBMET01548) were predicted to strongly bind to the SARS-CoV-2 M^pro^ active site according to the docking scores. Interestingly, while saquinavir metabolites are inactive against the HIV-1 protease (Noble and Faulds, 1996), they appear to bind to the SARS-CoV-2 M^pro^ binding site with a more favourable predicted binding energy than saquinavir. This finding may be relevant for the pharmacokinetics and dosing of this antiviral for the treatment of COVID-19. Indeed, saquinavir was shown to be inactive against the M^pro^, as recently reported in the COVID-19 open-data portal of the National Institutes of Health (NIH^11^). However, it was identified as a putative inhibitor of the spike glycoprotein (Ramírez-Salinas et al., 2020), and lately confirmed as active (NIH^11^). If experimentally confirmed, the potential activity of saquinavir metabolites on both the spike glycoprotein and the M^pro^ would result in beneficial effects on COVID-19 patients.

## Discussion

The goal of the present study was to perform a systematic drug repurposing screening of compounds from the DrugBank database for their ability to bind to the SARS-CoV-2 main protease. The performed analyses allowed the selection of 22 drugs or experimental compounds, which, to the best of our knowledge, can be considered “novel”, as they had not yet been included in COVID-19 clinical trials at the time of manuscript submission. Notably, several DrugBank compounds already reported as active against the SARS-CoV-2 M^pro^ scored among the top-ranked ligands, suggesting that the implemented protocol is able to correctly identify active ligands. Moreover, retrospective analyses were also performed on an additional set of known SARS-CoV-2 M^pro^ inhibitors, not included in the screened database, thus allowing to further validate the *in silico* workflow. Interestingly, many of the identified candidates could be able to combine a potential antiviral activity with other activities, e.g., antithrombotic or anti-inflammatory activity, due to their activity on the primary target and therapeutic indication. Such “polypharmacological” behavior, if confirmed, would make the identified candidate drugs extremely attractive to be further evaluated for COVID-19 treatment. Indeed, a single molecule would be able to concurrently exert an antiviral activity and mitigate or abolish COVID-19 comorbidities, the severity of which often leads to patient death. Therefore, in this study the importance of these repurposed molecules is also discussed from a polypharmacologic perspective. Furthermore, we identified a number of drug metabolites that appeared to be stronger binders of the SARS-CoV-2 M^pro^ than the parent drugs (e.g., saquinavir and ritonavir metabolites). To the best of our knowledge, drug repurposing based on drug (major) metabolites represents a novel approach that may offer additional and valuable opportunities to repurpose candidate drugs through the modulation of *in vivo* pharmacokinetics. In addition, the in-depth analysis of currently available literature data, including cell-based results on SARS-CoV-2 infected cells, further substantiate the relevance of the potential M^pro^ inhibitors selected in this work. Given the drastic need for therapeutic options for COVID-19, our results can suggest some key drugs for repurposing.

## Data Availability

The original contributions presented in the study are included in the article/Supplementary Material, further inquiries can be directed to the corresponding author.

## Footnotes

1 World Health Organization (2020) Coronavirus disease (COVID-19) pandemic. https://www.who.int/emergencies/diseases/novel-coronavirus-2019 [Accessed November 23, 2020].

2 Pfizer Inc. (2020) Pfizer and Biontech to submit emergency use authorization request today to the U.S. FDA for Covid-19 vaccine. https://www.pfizer.com/news/press-release/press-release-detail/pfizer-and-biontech-submit-emergency-use-authorization [Accessed November 23, 2020].

3 EMA (2020) https://www.ema.europa.eu/en/news/ema-recommends-first-covid-19-vaccine-authorisation-eu [Accessed December 21, 2020].

4 Clinicaltrials.gov (2020) COVID-19. https://clinicaltrials.gov/ct2/results?cond=COVID-19&term=&cntry=&state=&city=&dist= [Accessed November 6, 2020].

5 Clinicaltrials.gov (2020) Remdesivir. https://www.clinicaltrials.gov/ct2/results?term=remdesivir&cond=covid-19&Search=Apply&recrs=b&recrs=a&recrs=f&recrs=d&age_v=&gndr=&type=&rslt= [Accessed November 25, 2020].

6 World Health Organization (2020) Coronavirus disease (COVID-19): Hydroxychloroquine. https://www.who.int/news-room/q-a-detail/coronavirus-disease-covid-19-hydroxychloroquine [Accessed November 6, 2020].

7 National Institutes of Health (2020) COVID-19 - OpenData Portal. https://opendata.ncats.nih.gov/covid19/databrowser [Accessed December 21, 2020].

8 National Institutes of Health (2020) COVID-19 - OpenData Portal – Argatroban. https://opendata.ncats.nih.gov/covid19/databrowser?q=argatroban [Accessed December 22, 2020].

9 National Institutes of Health (2020) COVID-19 - OpenData Portal – Ritonavir. https://opendata.ncats.nih.gov/covid19/databrowser?q=Ritonavir [Accessed December 22, 2020].

10 National Institutes of Health (2020) COVID-19 - OpenData Portal – Lopinavir. https://opendata.ncats.nih.gov/covid19/databrowser?q=lopinavir [Accessed December 22, 2020].

11 National Institutes of Health (2020) COVID-19 - OpenData Portal – Saquinavir. https://opendata.ncats.nih.gov/covid19/databrowser?q=saquinavir [Accessed December 22, 2020].
