# Supplementary material for: Drug Repurposing and Polypharmacology to Fight SARS-CoV-2 Through Inhibition of the Main Protease
Source: Front Pharmacol. 2021 Feb 22;12:636989. doi: 10.3389/fphar.2021.636989 (PMC7938350; doi:10.3389/fphar.2021.636989)
Supplement: Supplementary file 1 [file DataSheet1.docx]

Supplementary Material

Drug repurposing and polypharmacology to fight SARS-CoV-2 through inhibition of the main protease

Luca Pinzi^1^, Annachiara Tinivella^1, 2^, Fabiana Caporuscio^1^, Giulio Rastelli^1^*

^1^ Molecular Modelling and Drug Design Lab, Life Sciences Department, University of Modena and Reggio Emilia, Modena, Italy.

^2^ Clinical and Experimental Medicine PhD Program, University of Modena and Reggio Emilia, Modena, Italy.

*** Correspondence:**Prof. Giulio Rastelli, Life Sciences Department, University of Modena and Reggio Emilia, Via Campi 103, 41125 Modena, Italy. giulio.rastelli@unimore.it

**TABLE OF CONTENTS**

Figures

Figure S1 ………………………………………………………………........................................... S3

Tables

Table S1 .…………………………………………………………………........................................ S5

Table S2 ……………………………………………………………………………………………. S6

**References** ..………………………………………………………………....................................... S7

**Figure S1**: Chemical structures of the identified candidates.


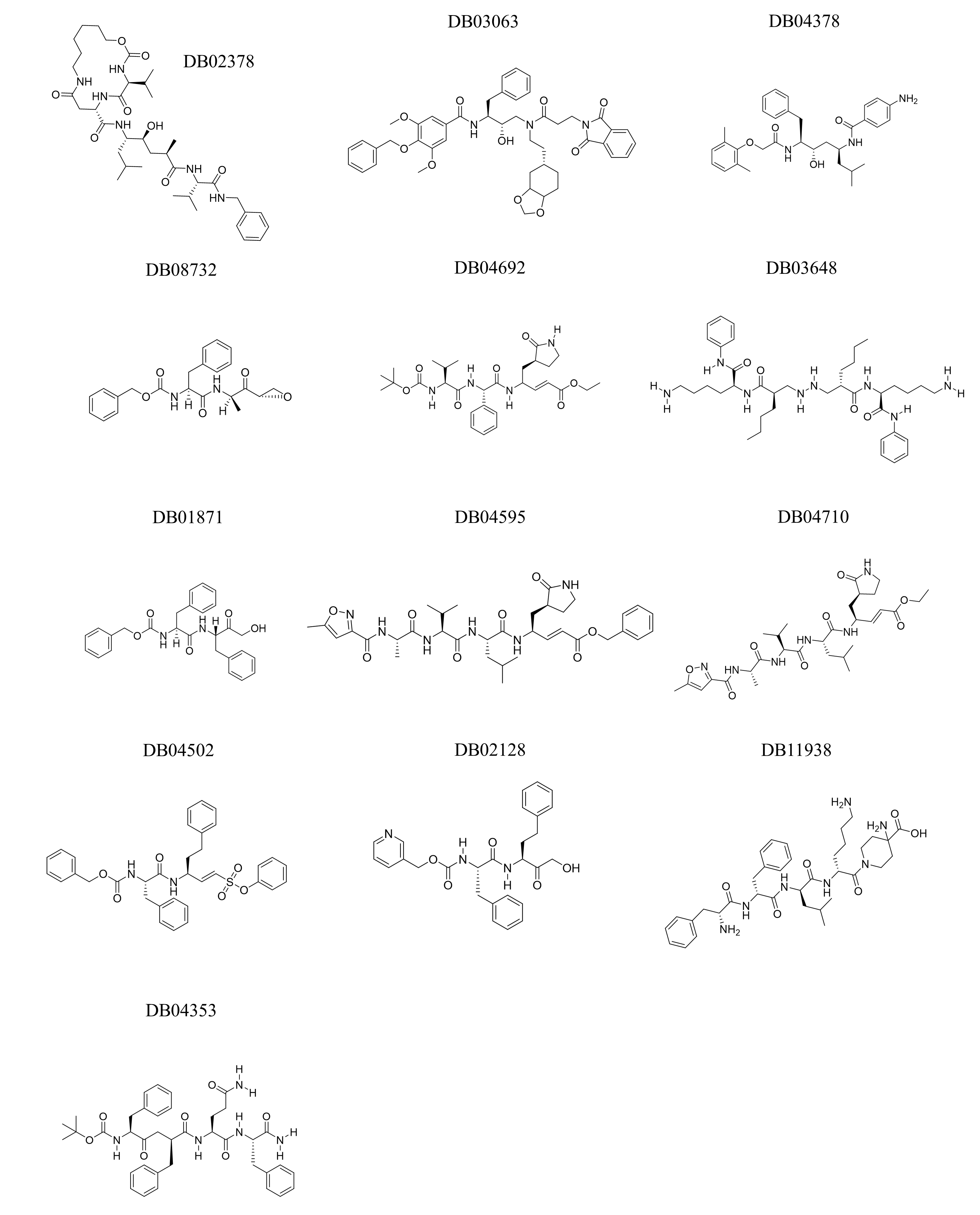


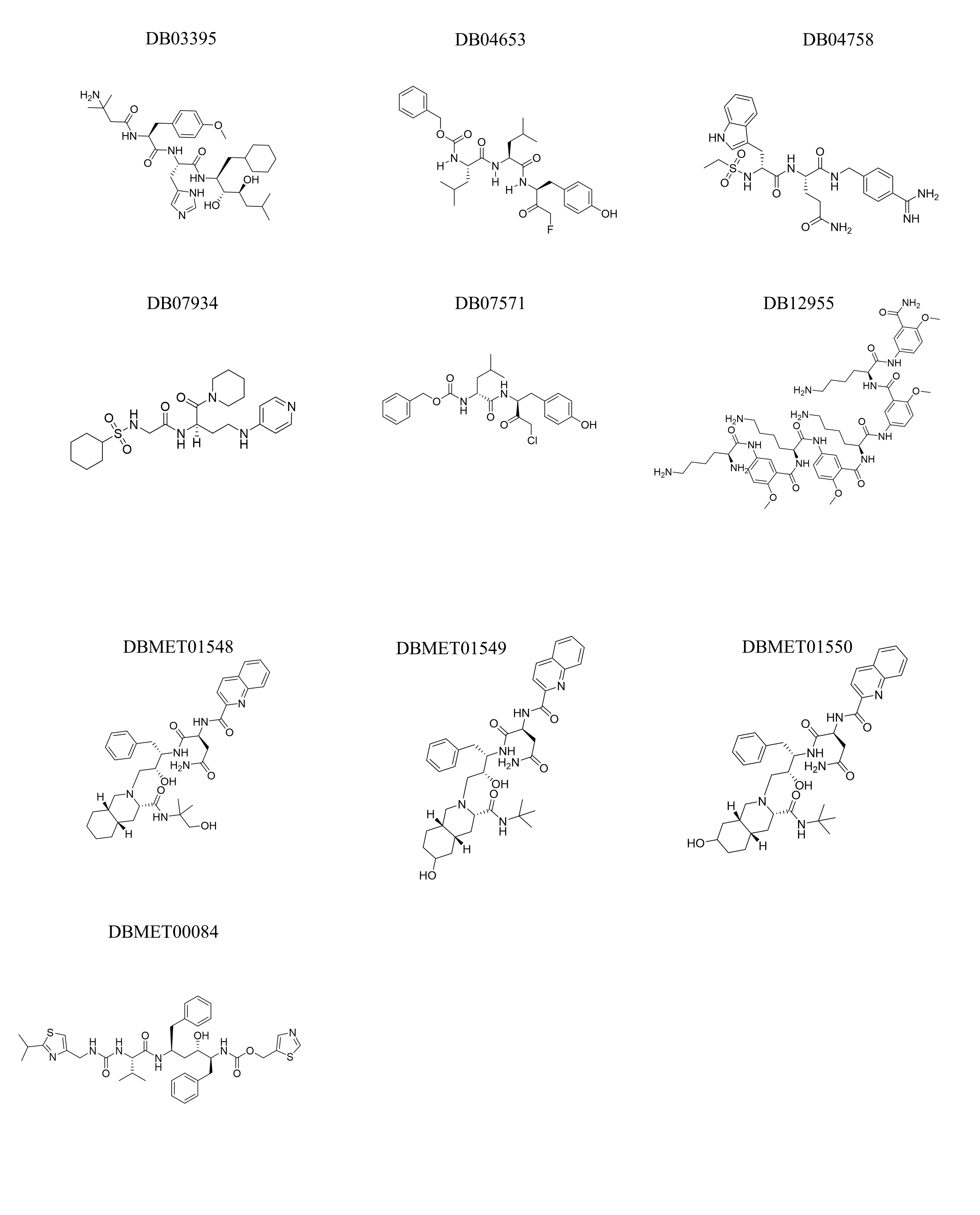


**Table S1**. Selected ligands with already reported activity data on the SARS-CoV-2 M^pro^ used in the retrospective validation of the structure-based virtual screening protocol.

| ***DrugBank ID*** | ***Compounds Name*** | ***Bibliographic References*** | ***Scores (kcal/mol)*** | | | ***RANK #*** | | | ***SARS-CoV-2 M^pro^ IC_50_ (μM)^*^*** |
| --- | --- | --- | --- | --- | --- | --- | --- | --- | --- |
|  |  |  | ***Glide*** | ***BEAR GB*** | ***BEAR PB*** | ***Glide*** | ***BEAR GB*** | ***BEAR PB*** |  |
| DB12610 | Ebselen | Jin et al., 2020 | / | / | / | / | / | / | 0.67 |
| **DB04595** | **N3; PRD_002214** | Jin et al., 2020 | **-9.9** | **-58.4** | **-35.8** | **42** | **292** | **151** | **16.77*** |
| DB00822 | Disulfiram | Jin et al., 2020 | -4.3 |  |  | 10127 |  |  | 9.35 |
| DB12129 | Tideglusib | Jin et al., 2020 | -6.4 |  |  | 5230 |  |  | 1.55 |
| DB09010 | Carmofur | Jin et al., 2020 | -6.3 |  |  | 6209 |  |  | 1.82 |
| DB05448 | PX-12 | Jin et al., 2020 | -5.3 |  |  | 10021 |  |  | 21.39 |
| DB06290 | Simeprevir (24) | Ma et al., 2020 | -5.9 |  |  | 9306 |  |  | 13.74 |
| DB08873 | Boceprevir (28) | Ma et al., 2020 | -8.2 | -59.7 | -39.1 | 384 | 264 | 89 | 4.13 |
| DB14760 | Narlaprevir (29) | Ma et al., 2020 | -8.2 | -66.9 | -40.3 | 423 | 101 | 66 | 5.73 |
| DB07558 | Calpain inhibitor I (ALLN) (59) | Ma et al., 2020 | -8.7 | -41.5 | -29.4 | 193 | 1221 | 480 | 8.60 |
| DB05102 | AG7088; Rupintrivir (65) | Ma et al., 2020 | -9.4 | -64.1 | -34.4 | 78 | 155 | 192 | >20 |
|  | Shikonin | Jin et al., 2020 | -5.9 |  |  |  |  |  | 15.75 |
|  | MG-132 (43) | Ma et al., 2020 | -9.2 | -63.1 | -42.7 |  |  |  | 3.90 |
|  | Calpeptin (56) | Ma et al., 2020 | -8.6 | -51.3 | -29.6 |  |  |  | 10.69 |
|  | Calpain inhibitor III (MDL28170) (57) | Ma et al., 2020 | -7.8 | -47.6 | -26.9 |  |  |  | >20 |
|  | Calpain inhibitor VI (58) | Ma et al., 2020 | -7.4 | -44.4 | -31.9 |  |  |  | >20 |
|  | MG-115 (60) | Ma et al., 2020 | -9.4 | -54.2 | -23.4 |  |  |  | 3.14 |
|  | Calpain inhibitor II (ALLM) (61) | Ma et al., 2020 | -7.9 | -52.7 | -35.6 |  |  |  | 0.97 |
|  | Calpain inhibitor XII (62) | Ma et al., 2020 | -7.6 | -52.2 | -37.8 |  |  |  | 0.45 |
|  | PSI (63) | Ma et al., 2020 | -7.7 | -63.3 | -40.5 |  |  |  | 10.38 |
|  | GC-376 (64) | Ma et al., 2020 | -9.5 | -50.9 | -32.2 |  |  |  | 0.03 |
|  | 11a | Dai et al., 2020 | -10.7 | -62.7 | -50.9 |  |  |  | 0.053 |
|  | 11b | Dai et al., 2020 | -8.9 | -48.7 | -32.16 |  |  |  | 0.04 |

Note:

* Activity values expressed as a mean of EC_50_. Compound DB04595, which is the SARS-CoV-2 M^pro^ inhibitor co-crystallized in the 6LU7 PDB complex is shown in bold. Compounds that were ranked according to their Glide score (“*Docking Score*”) out of the top 2000 compounds are shown in red.

**Table S2**. Reported crystal structures of the repurposed candidate compounds.

| ***DrugBank ID*** | ***Compound Name*** | ***PDB Codes*** |
| --- | --- | --- |
|  |  |  |
| **DB01871** | CRUZ-1; EXPT02989 | 1ME4 (Huang et al., 2003) |
| **DB02128** | CRUZ-2; EXPT02467 | 1ME3 (Huang et al., 2003) |
| **DB02378** | MMI-175; EXPT02196 | 1XS7 (Ghosh et al., 2005) |
| **DB03063** | EH58; EXPT01332 | 1LF3 (Asojo et al., 2003) |
| **DB03648** | EXPT00713 | 1RL4 (Robien et al., 2004) |
| **DB04378** | RS370; EXPT02746 | 1LF2 (Asojo et al., 2002) |
| **DB04502** | WRR-204; EXPT03235 | 1EWO (Brinen et al., 2003) |
| **DB04595** | N3; PRD_002214 | 6LU7 (Jin et al., 2020) |
| **DB04692** | I2 | 2D2D (Yang et al., 2005) |
| **DB08732** | WRR-183 | 2OP9 (Goetz et al., 2007) |
| **DB04710** | N1 | 1WOF; 2AMP (Yang et al., 2005) |
| **DB04353** | QF34; EXPT02729 | 1IZH; 1IZI (Weber et al., 2002) |
| **DB04653** | Calpain inhibitor IV; ZLLYCH_2_F | 1ZCM (Li et al., 2006) |
| **DB04758** | Ethylsulfonamide-D-Trp-Gln-p-aminobenzamidine | 1WUN (Kadono et al., 2005) |
| **DB07571** | Z-LY-CMK | 2FZS (Engh et al., 1996) |
| **DB07934** | BM51.1011 | 1UVS (Zhang et al., 2020) |

References

Asojo, O. A., Afonina, E., Gulnik, S. V., Yu, B., Erickson, J. W., Randad, R., et al. (2002). Structures of Ser205 mutant plasmepsin II from Plasmodium falciparum at 1.8 Å in complex with the inhibitors rs367 and rs370. *Acta Crystallogr., Sect. D: Biol. Crystallogr.* 58, 2001–2008. doi:10.1107/S0907444902014695.

Asojo, O. A., Gulnik, S. V., Afonina, E., Yu, B., Ellman, J. A., Haque, T. S., et al. (2003). Novel uncomplexed and complexed structures of plasmepsin II, an aspartic protease from Plasmodium falciparum. *J. Mol. Biol.* 327, 173–181. doi:10.1016/S0022-2836(03)00036-6.

Brinen, L. S., Gillmor, S. A., and Fletterick, R. J. Crystal structure of cruzain bound to WRR-204. Protein Data Bank (2003) doi:10.2210/pdb1EWO/pdb.

Dai, W., Zhang, B., Jiang, X.-M., Su, H., Li, J., Zhao, Y., et al. (2020). Structure-based design of antiviral drug candidates targeting the SARS-CoV-2 main protease. *Science* 368, 1331–1335. doi:10.1126/science.abb4489.

Engh, R. A., Brandstetter, H., Sucher, G., Eichinger, A., Baumann, U., Bode, W., et al. (1996). Enzyme flexibility, solvent and “weak” interactions characterize thrombin-ligand interactions: Implications for drug design. *Structure* 4, 1353–1362. doi:10.1016/S0969-2126(96)00142-6.

Ghosh, A. K., Devasamudram, T., Hong, L., Dezutter, C., Xu, X., Weerasena, V., et al. (2005). Structure-based design of cycloamide-urethane-derived novel inhibitors of human brain memapsin 2 (β-secretase). *Bioorganic Med. Chem. Lett*. 15, 15–20. doi:10.1016/j.bmcl.2004.10.084.

Goetz, D. H., Choe, Y., Hansell, E., Chen, Y. T., McDowell, M., Jonsson, C. B., et al. (2007). Substrate specificity profiling and identification of a new class of inhibitor for the major protease of the SARS Coronavirus. *Biochemistry* 46, 8744–8752. doi:10.1021/bi0621415.

Huang, L., Brinen, L. S., and Ellman, J. A. (2003). Crystal structures of reversible ketone-based inhibitors of the cysteine protease cruzain. *Bioorganic Med. Chem.* 11, 21–29. doi:10.1016/S0968-0896(02)00427-3.

Jin, Z., Du, X., Xu, Y., Deng, Y., Liu, M., Zhao, Y., et al. (2020). Structure of Mpro from SARS-CoV-2 and discovery of its inhibitors. *Nature* 582, 289–293. doi:10.1038/s41586-020-2223-y.

Kadono, S., Sakamoto, A., Kikuchi, Y., Oh-eda, M., Yabuta, N., Yoshihashi, K., et al. (2005). Structure-based design of P3 moieties in the peptide mimetic factor VIIa inhibitor. *Biochem. Biophys. Res. Commun.* 327, 589–596. doi:10.1016/j.bbrc.2004.12.042.

Li, Q., Hanzlik, R. P., Weaver, R. F., and Schönbrunn, E. (2006). Molecular mode of action of a covalently inhibiting peptidomimetic on the human calpain protease core. *Biochemistry* 45, 701–708. doi:10.1021/bi052077b.

Ma, C., Sacco, M. D., Hurst, B., Townsend, J. A., Hu, Y., Szeto, T., et al. (2020). Boceprevir, GC-376, and calpain inhibitors II, XII inhibit SARS-CoV-2 viral replication by targeting the viral main protease. *Cell Res.* 30, 678–692. doi:10.1038/s41422-020-0356-z.

Robien, M. A., Nguyen, K. T., Kumar, A., Hirsh, I., Turley, S., Pei, D., et al. (2004). An improved crystal form of Plasmodium falciparum peptide deformylase. *Protein Sci.* 13, 1155–1163. doi:10.1110/ps.03456404.

Weber, J., Mesters, J. R., Lepšík, M., Prejdová, J., Švec, M., Šponarová, J., et al. (2002). Unusual binding mode of an HIV-1 protease inhibitor explains its potency against multi-drug-resistant virus strains. *J. Mol. Biol.* 324, 739–754. doi:10.1016/S0022-2836(02)01139-7.

Yang, H., Xie, W., Xue, X., Yang, K., Ma, J., Liang, W., et al. (2005). Design of wide-spectrum inhibitors targeting coronavirus main proteases. *PLoS Biol.* 3, e324. doi:10.1371/journal.pbio.0030324.

Zhang, L., Lin, D., Sun, X., Curth, U., Drosten, C., Sauerhering, L., et al. (2020). Crystal structure of SARS-CoV-2 main protease provides a basis for design of improved α-ketoamide inhibitors. *Science* 368, 409–412. doi:10.1126/science.abb3405.
